# Desert lizards modulate nutritional responses to match seasonal biological needs

**DOI:** 10.1098/rsos.251690

**Published:** 2026-01-07

**Authors:** Mihir Joshi, Avichal Tatu, Dror Hawlena, David Raubenheimer, Maria Thaker

**Affiliations:** 1 Centre for Ecological Sciences, Indian Institute of Science, Bengaluru, Karnataka, India; 2 School of Biosciences, University of Melbourne, Melbourne, Victoria, Australia; 3 Department of Ecology, Evolution and Behavior, Hebrew University, Jerusalem, Israel; 4 Department of Charles Perkins Centre, The University of Sydney, Sydney, New South Wales, Australia

**Keywords:** nutritional ecology, diet, faecal composition, elemental analysis, omnivory, herbivory

## Abstract

Animals in extreme environments such as deserts experience severe seasonal fluctuations in abiotic conditions and resources. Under these conditions, they must obtain macronutrients in optimal amounts and ratios to meet their nutritional requirements, which themselves vary with seasonal changes in physiological and life-history processes. To understand whether nutritional intakes and retention align with key life-history events across seasons, we examined the nutritional ecology of the desert-dwelling lizard, *Saara hardwickii*. We first measured seasonal variation in the quantity and quality of plants available for these herbivorous lizards. By combining field observations of foraging behaviour with elemental analysis, we determined the carbon (C) and nitrogen (N) levels in dietary intakes and faecal matter across seasons. Intake C : N was lowest and faecal C : N was highest in June, reflecting greater proportional consumption and retention of N, likely meeting increased protein demands during breeding. Conversely, dietary C : N and faecal %N were highest in October, indicating greater consumption and retention of C prior to hibernation. Surprisingly, these putative herbivores consumed insects only around the breeding season, despite their year-round availability. Overall, we show that *S. hardwickii* use both behavioural diet choice and post-ingestive physiology to match seasonal nutritional needs by differentially consuming and retaining nutrients in an extreme environment.

## 1 Introduction

Extreme habitats, such as deserts, are marked by harsh and unpredictable seasonal fluctuations in environmental conditions, to which animals must adapt. Animals inhabiting such environments experience extreme temperatures, irregular water availability and patchy spatio-temporal distribution of resources that also vary in quality and availability [1,2]. Extreme heat or cold can shift the activity budgets of animals, forcing them to forage in narrow time windows with favourable conditions [3]. Animals also shift their diet to match extreme resource fluctuations. For example, goitered gazelles, *Gazella subgutturosa*, in the arid regions of western China shift from consuming mostly forbs in spring to mostly trees before winter due, principally, to the unavailability of forbs during colder months [4]. Prolonged periods of resource scarcity and stressful conditions can also force animals to hoard resources while they are available and/or reduce their metabolic rates when they are unable to forage [5]. Thus, animals in habitats such as deserts must adjust their foraging strategies to highly seasonal patterns of resource availability, which may pose a challenge in meeting their nutritional requirements and achieving optimal fitness outcomes.

Nutritional requirements of animals are determined by underlying biological processes that vary across life stages and contexts. Production-related processes such as growth and reproduction are highly dependent on amino acids derived from proteins as raw materials, whereas carbohydrates are the main fuel source for maintenance and energy production [6]. Animals are both sensitive and responsive to such changes in nutritional demands. For instance, during the breeding season, the proportion of body protein in pregnant females of northern ungulates increases by minimizing the metabolic breakdown of proteins in this season to support breast-milk production [7]. Many vertebrates that hibernate in winter need to consume and store enough carbohydrates or lipids to survive without foraging for months. For example, grizzly bears double their fat reserves by consuming foods rich in lipids and proteins before hibernation [8]. Seasonal shifts in such biological processes generate nutritional demands specific to the season. Therefore, the performance and survival of animals depend on how well they can meet their season-specific nutritional needs. While the nutritional ecology of seasonal foraging has been studied in cold temperate and tropical habitats (e.g. [9,10]), how desert vertebrates compensate for seasonally changing nutritional needs is less understood.

Animals can meet their nutritional requirements by ingesting foods of different nutritional composition or by regulating absorption and retention of nutrients post-ingestively. For instance, many marine herbivorous fishes are omnivores during their juvenile phase to meet the protein requirements of growth [11]. Meeting nutritional requirements through such dietary changes is effective when animals have access to either a balanced food that has an optimal ratio of required macronutrients or complementary foods that can be combined to obtain optimum nutrition [6,12,13]. Additionally, downstream physiological processes can selectively influence the absorption of resources by modifying the rates at which different macronutrients are absorbed or voided from the gut [6,14]. For example, amphibians change their gut morphology and modulate nutrient transport rates to optimize gut performance under varying ecological conditions [15]. Although it is apparent that animals can satisfy their nutritional needs either behaviourally through foraging decisions or post-ingestively, understanding how these two mechanisms interact to affect the trophic function of free-ranging vertebrates is challenging, especially in arid ecosystems that change substantially across seasons.

To address these gaps, we studied the nutritional ecology of Indian spiny-tailed lizards, *Saara hardwickii*, that inhabit the Thar desert in the northwestern region of India, along with parts of Pakistan and Afghanistan [16,17] (electronic supplementary material, figure S1). These agamid lizards are active from February to October and hibernate in burrows from November to February [18]. Within this activity period, they mate during April–May and lay eggs from the end of May to July. Foraging successfully in the Thar desert is challenging since the availability of food resources varies greatly across seasons, especially for adult lizards that are considered predominantly herbivorous with some records of occasional insectivory [19–21]. Despite the harsh desert conditions and challenging foraging requirements, these lizards thrive, with population density reaching as high as 700 individuals/hectare in parts of the landscape that have a high plant density (M.J., unpublished). This species is also relatively large (snout-to-vent length = 19.88 cm; M.J., unpublished data), making them a good system for field-based quantification of nutritional intakes. Given their life history, this species is expected to require different ratios of macronutrients across the annual cycle, which include meeting the nutritional demands of reproduction and hibernation. How these lizards use behaviour and/or physiology to meet these nutritional demands under seasonal environmental constraints is largely unknown.

To this end, we measured the elemental composition of all the plants available and consumed by the spiny-tailed lizards in their habitat across four months that represent four different seasonal conditions. To determine the composition and nutritional quality of lizard diets, we conducted focal observations and recorded amounts and types of foods consumed across four seasons. We combined these foraging data with elemental analyses of dietary components to estimate the composition (in terms of carbon (C) and nitrogen (N)) of lizard dietary intake. Elements provide a proxy for diet macronutrient composition [22,23] that can be readily tracked in their passage through organisms and are thus useful for constructing nutrient budgets in an ecological context [24]. We also measured the elemental composition of faecal matter to determine whether these lizards exhibit differences in post-ingestive regulation of nutrients across seasons. We predict that lizards will consume and retain higher amounts of N (principally from proteins) in the breeding season and higher amounts of C (principally from carbohydrates and lipids) during the pre-hibernation season to build fat stores. With the first measures of nutritional ecology for this species, this study furthers our understanding of how nutritional needs drive behavioural and physiological responses in vertebrates, with the potential to shape trophic interactions in desert ecosystems.

## 2 Methods

### 2.1 Study site

**Figure 1:**
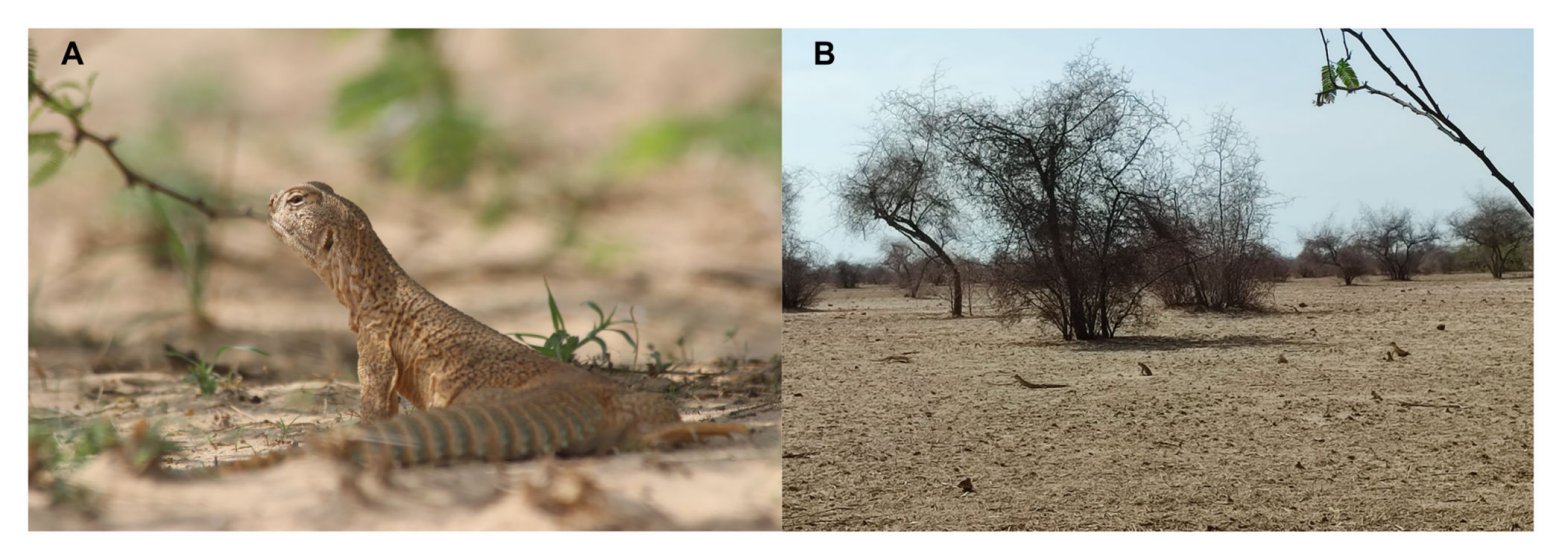
(A) Indian spiny-tailed lizard (*Saara hardwickii*) adult. (B) *S. hardwickii* adults basking and foraging in their natural habitat in Jorbeer-Gadhwala Conservation Reserve, Bikaner, Rajasthan, India.

The study was carried out from April to October of 2022 in Jorbeer-Gadhwala Conservation Reserve located in the Thar desert of northwestern India (figure 1). The Thar experiences extremely hot and arid summers (April to June) when air temperatures can reach 51°C and cold winters (November to February) when the air temperature can drop to 0°C. This region receives erratic bouts of monsoon rain (100–450 mm yearly) during the months of July and August. Jorbeer-Gadhwala Conservation Reserve constitutes large expanses of sandy plains, which are the main habitats for *S. hardwickii*, interspersed with sand dunes. Few species of shrubs, herbs and grasses that grow in these sandy plains vary in species richness and abundance across seasons, creating a fluctuating resource pool for herbivores inhabiting this landscape.

We set up 20 plots of 20 × 20 m each for plant biomass and dietary intake sampling. These plots were first selected visually to have similar vegetation, which was later confirmed by the estimation of plant biomass and community composition. This plot size was selected because *S. hardwickii* typically range no more than 10–15 m away from their burrows while foraging (M.J., unpublished). We selected plots that were at least 100 m apart to ensure that we sampled a different set of lizards in each plot. We also recorded the number of individuals in each plot to ensure that the foraging behaviour was not affected by the plot-level lizard density (which ranged from 5 to 11 individuals). Sampling was repeated in all 20 plots in April (early summer), June (late summer, breeding), August (monsoon, post-breeding) and October (early winter, pre-hibernation).

### 2.2 Plant biomass and species composition

**Figure 2:**
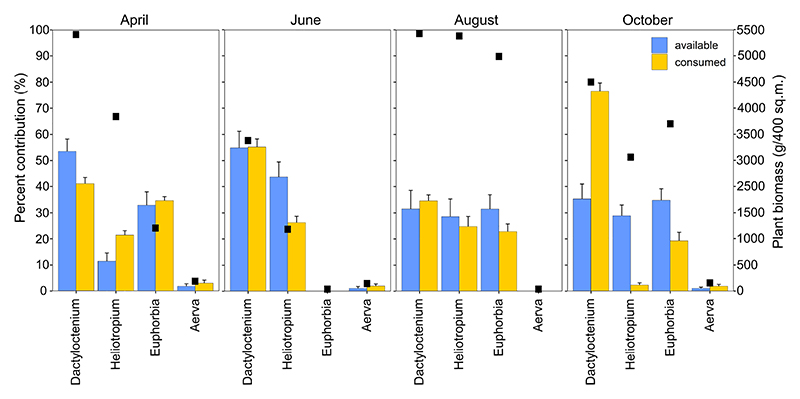
Percent contribution of main food plants to available resources (blue bars) and *S. hardwickii* diet (yellow bars). Black squares represent the absolute biomass values of each plant species across four seasons.

To establish plant biomass and species composition, we set up 20 smaller quadrats of 1 m^2^ in each of the larger sampling plots (electronic supplementary material, figure S1). The quadrats were selected systematically in a cross and photographed in a single day across plots. Grasses and herbs within the quadrats were identified to the genus level manually from the photos. Since fewer than 10 plant species were found growing on the sandy plains in any given season, we were able to reliably identify food plants based on their morphology. These images were later superimposed with a 5 × 5 frame using ImageJ, and the percentage area covered by each plant species was visually estimated for all quadrats. After the quadrats were photographed, we removed all the plants in the quadrat and weighed them using a Pesola spring balance in the field. Since the wet and dry biomass of plants was highly correlated (electronic supplementary material, figure S2), we used the wet biomass values measured at the scale of each quadrat and converted them into available biomass in the 400 m^2^ area of the sampling plots (figure 2, electronic supplementary material, figure S2). We calculated the percentage contribution of each species to the plant biomass in the season as %*M_i_* = ∑ (*M_t_* * %*S_i_*), where *M_t_* is the average total biomass per plot each season and %*S_i_* is the average per cent cover of species *i* per plot for the season (figure 2).

### 2.3 Dietary intakes

To estimate the dietary intakes of *S. hardwickii*, we conducted focal observations to record the number of bites that adult lizards took from each plant species as well as the number of insects consumed throughout the entire feeding day. These focal observations were repeated in a different plot each day within a season. We also calculated the amount of each food item consumed across seasons using bite count data and the leaf mass for each species. We plucked and weighed the leaves of all plant species consumed by *S. hardwickii*. For bite counts, we set up an observation hide approximately 10 m from the plot and carried out behavioural observations of individuals from a single plot from 07.00 to 19.00. We followed different individuals within each plot for 15 min durations throughout the day, with a 5 min break between each bout of focal observations. We sampled both adult males and females within the plot since individuals were unmarked (as permits to capture and mark lizards were not available due to the COVID-19 lockdowns) and thus, plots are our sampling units for analyses (20 intake values/season).

To estimate the elemental composition of intakes, we collected all plant species available to these lizards across seasons. Plant samples were collected in ziplock bags and sun-dried on the day of collection to avoid fungal contamination. We collected leaves from more than 10 individuals of each plant species and measured the elemental composition from these leaves. We also hand-collected the insect species observed to be consumed by these lizards and manually identified them to the order level (mainly termites in June and true bugs in August). We did not expect insects to be a major part of the diet of this species, as it was hitherto reported to be an herbivore. However, during data analysis, we discovered a significant contribution of insects to lizard diets, at which point it was too late to estimate insect abundance while observing lizard foraging behaviour. To estimate the elemental composition of excreta, we collected fresh faecal pellets in ziplock bags after each observation day. All the pellets from a single plot were sun-dried and pooled for analysis. Plant and insect samples were stored at 4°C to prevent fungal contamination. Dry faecal pellets did not show signs of moulding, so they were stored at room temperature until they were transferred to the laboratory for elemental analysis.

### 2.4 Elemental composition

We oven-dried all plant, insect and faecal samples at 40°C for 24 h. Dried samples were crushed into a fine powder using a motorized grinder (plants and faecal matter) or mortar and pestle (insects) and analysed in the elemental analyser (Elementar Analysensysteme GmbH, Germany), which was calibrated with sulfanilamide standards. The carbon and nitrogen content (%C, %N) values for the plant and insect samples were used to further calculate the composition of lizard intakes.

To estimate the elemental composition of lizard intakes, we calculated the total C and N consumed by these lizards across seasons. For these calculations, we assumed that a uniform amount of biomass was consumed with each bite and used the average leaf mass measured for each species. Total carbon consumption per day (C_T_) was calculated using the formula C_T_ = ∑ (*B_i_* * %*C_i_*), where *B_i_* is the number of bites of each food item taken and %*C_i_* is the proportion of carbon by mass for the respective food item. Similarly, total nitrogen consumed per day (N_T_) was calculated as: N_T_ = ∑ (*B_i_* * %*N_i_*), where %*N_i_* is the proportion of nitrogen by mass for each food item. We used the average daily total C and N consumed by the lizards to calculate the carbon to nitrogen ratios of lizard intakes (C_T_/N_T_).

To understand the contributions of each food item to the diet composition of these lizards across seasons, we calculated the per cent C, per cent N and per cent mass for each dietary item each season. We also calculated the percentage of C and N by mass in their diet and faecal matter across seasons to determine the shifts in consumed as well as voided nutrients.

### 2.5 Data analysis

To assess whether selection of food plants across seasons deviated from what is expected as a result of random foraging, we simulated null diets for *S. hardwickii* in each season based on network-based null models using ‘econullnetr’ [25]. These null diets, generated with the ‘generate_null_net’ command, represent lizard diets as a function of resource density alone. We then compared the null diet with observed interactions (i.e. proportions of food plants in lizard diet) using ‘species-level’ bipartite statistics to estimate the extent to which diet choice deviated from random in each season. We report standardized effect sizes (SES), observed interaction strength and null estimates for all four seasons. To determine the variation in dietary intakes across seasons, we ran three linear mixed-effect models (LMMs) with normal distribution for intake C : N (to examine seasonal shifts in dietary intake composition), total daily C and total daily N (to test whether C or N intakes drove the changes in the relative ratio of intake C : N) from all the plots across four seasons using the package *lme4* [26]. In these models, season was the fixed factor and ‘plot number’ was the random effect to control for the potential between-plot differences since the same set of plots were sampled across seasons. We also examined whether the elemental ratios in faecal samples varied across seasons using a LMM with season as a fixed factor and ‘plot number’ as a random effect. Finally, we ran linear models (LM) to examine the differences in the proportion of carbon and nitrogen contributed by each dietary component in all the seasons. We ran a separate LM for %C and %N in each season, with diet component as the fixed factor. Where relevant, we ran post hoc pairwise comparisons (Tukey-adjusted). All statistical analyses were done using R (v. 3.6.3), and the tables of pairwise comparisons across seasons for all the models are reported in electronic supplementary material.

## 3 Results

We found that *S. hardwickii* could adjust their foraging behaviour as well as post-ingestive nutrient retention to match their seasonal nutritional requirements. Across seasons, these lizards predominantly consumed four plants in this landscape: *Dactyloctenium* sp., *Heliotropium* sp., *Euphorbia* sp. and *Aerva* sp. *Dactyloctenium* sp. was consistently the most abundant (31–55%) and consumed species (34–76% of the diet) across seasons, while *Aerva* sp. was the least abundant (1–2%) and consumed (1–4%) species across seasons (figure 2). Plants constituted the entire diet of *S. hardwickii* in April and October, and a major part of their diet in June and August. The selection of food plants deviated significantly from a null foraging choice based purely on resource density, indicating a preference for certain food plants across seasons. Interaction strength was higher than the null prediction across all seasons (April: SES = 2.01, observed = 0.42, null = 0.28 ± 0.07; June: SES = 1.83, observed = 0.39, null = 0.29 ± 0.08; August: SES = 1.47, observed = 0.27, null = 0.18 ± 0.06; October: SES = 1.91, observed = 0.4, null = 0.3 ± 0.07). Insects were only seen in their diet during June and August, which coincide with the breeding and post-breeding seasons for the species.

**Figure 3:**
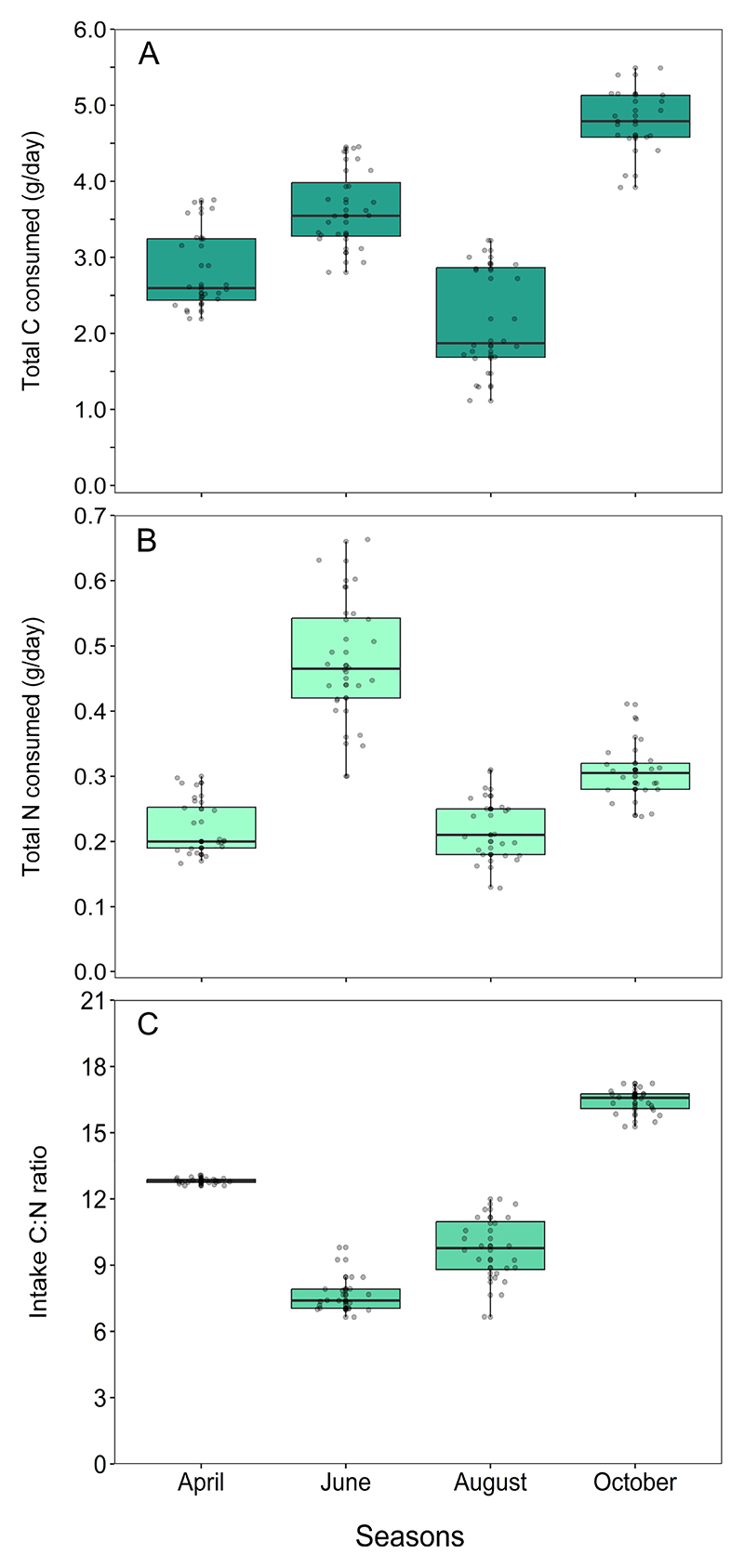
Total C (A), total N (B) and C : N ratios (C) of *S. hardwickii* intakes across four seasons. Box plots represent the median amount of nitrogen and carbon consumed per day along with the interquartile range. Vertical lines represent quartile 1 – 1.5 × interquartile range (IQR) and quartile 3 + 1.5 × IQR.

**Figure 4:**
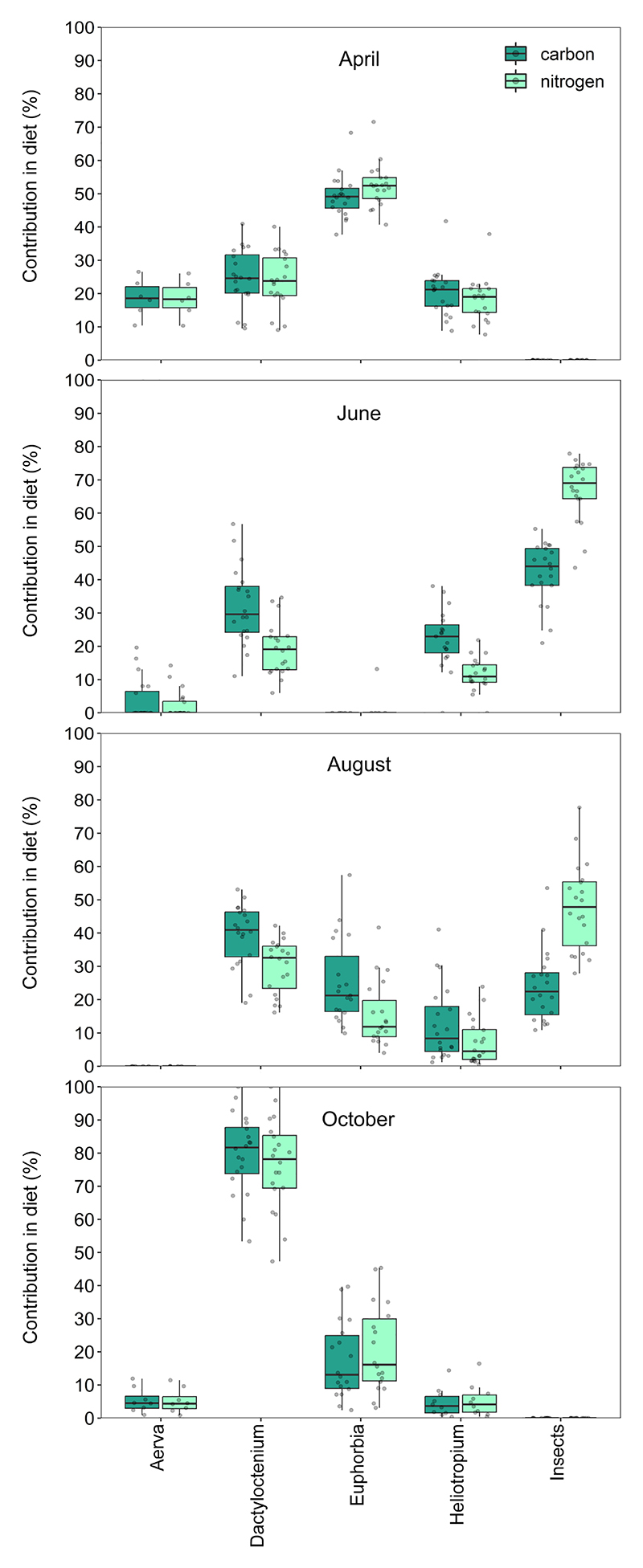
Per cent contribution of each dietary component to the total C (dark green) and total N (light green) consumed across four seasons. Box plots represent the median amount of nitrogen and carbon consumed per day along with the interquartile range. Vertical lines represent quartile 1 – 1.5 × IQR and quartile 3 + 1.5 × IQR.

We found that the total carbon (*χ*^2^ = 240.3, *p* < 0.001) and total nitrogen consumed (*χ*^2^ = 236.1, *p* < 0.001) varied significantly across seasons (figure 3A,B). Daily carbon consumed was higher in October than in April (*Z* = 11.30, *p* < 0.001), June (*Z* = 7.24, *p* < 0.001) and August (*Z* = 14.71, *p* < 0.001). This was followed by a higher carbon intake in June compared with April (*Z* = 4.06, *p* < 0.001) and August (*Z* = 7.48, *p* < 0.001). Daily carbon consumption in April was greater than in August (*Z* = 3.42, *p* = 0.004). From the diet analysis, we found that the lizards derived most of their dietary carbon from *Dactyloctenium* sp. in October (figure 4). In contrast, the daily nitrogen intake was higher in June compared with April (*Z* = 13.26, *p* < 0.001), August (*Z* = 13.36, *p* < 0.001) and October (*Z* = 8.84, *p* < 0.001). This excess nitrogen in June was mostly derived from insects (figure 4). Nitrogen intake in October, derived mostly from *Dactyloctenium* sp. (figure 4), was higher than that in April (*Z* = 4.42, *p* = 0.001) and August (*Z* = 4.52, *p* < 0.001), whereas intake levels did not differ between April and August (*Z* = 0.10, *p* = 0.996). See electronic supplementary material for percentage contributions of each diet component to total carbon, total nitrogen and mass of lizard diets across seasons (electronic supplementary material, table S2).

**Figure 5:**
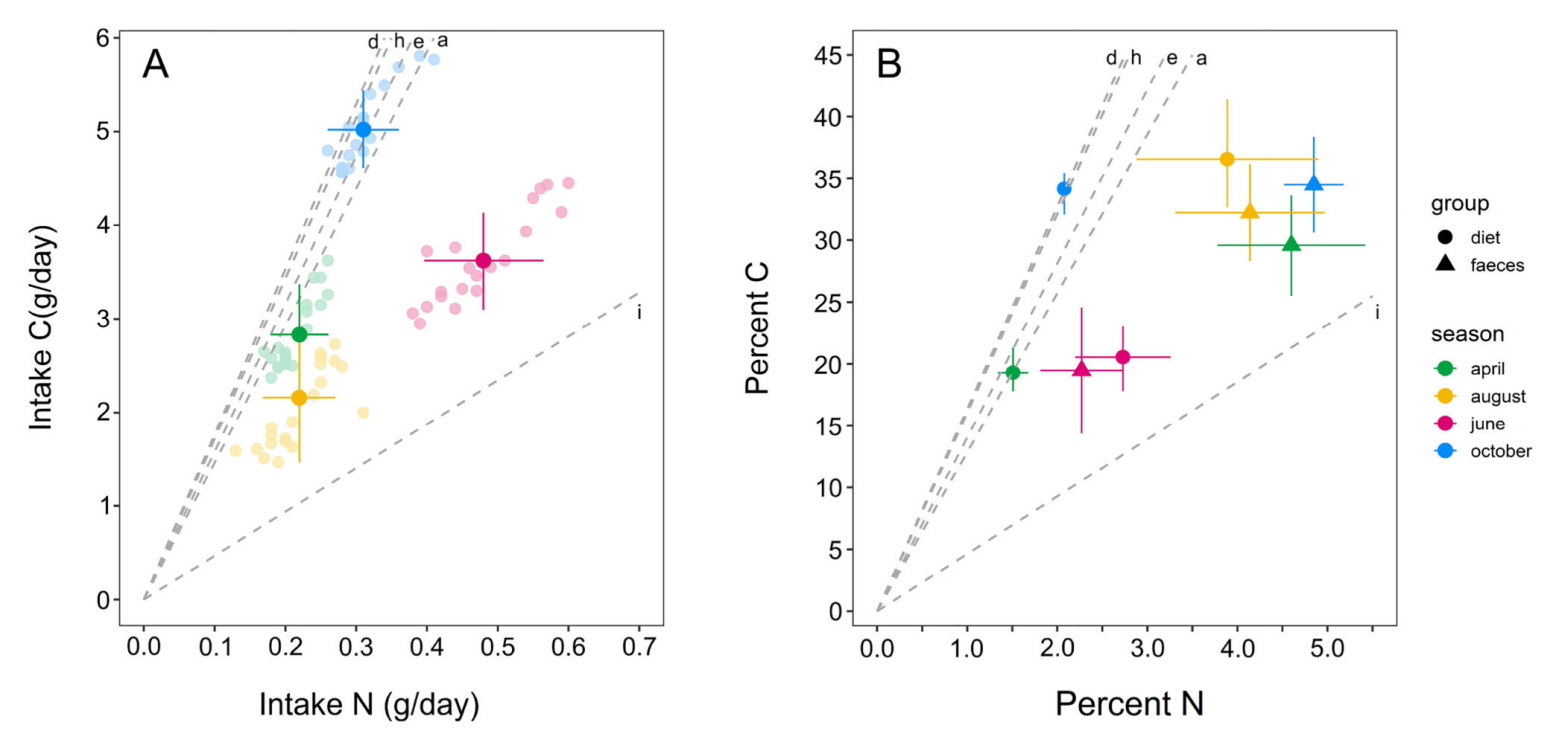
(A) Dietary intakes (mean ± s.d.) for *S. hardwickii* across four seasons visualized in a nutritional state space defined by total C and total N (in g) consumed per day. The smaller, lighter circles represent daily intake points for each season. (B) %C and %N of intakes and faecal matter (means ± s.d.) visualized in a nutritional state space. Grey dashed lines in both panels represent the mean C : N of the five dietary components consumed (d: *Dactyloctenium*, h: *Heliotropium*, e: *Euphorbia*, a: *Aerva*, i: insects), defining a nutrient state space for these lizards across seasons.

As expected from the seasonal differences in carbon and nitrogen consumed by the lizards, the C : N of intakes differed significantly across seasons (*χ*^2^ = 214.5, *p* < 0.001, figure 3 and figure 5A). Intake C : N was significantly higher in October, the early-winter season, compared with April (*Z* = 13.49, *p* < 0.001), June (*Z* = 32.64, *p* < 0.001) and August (*Z* = 24.98, *p* < 0.001), indicating an increased proportion of carbon in the diet (figure 3, 5A,B). Intake C : Ns in June, which is the peak breeding season, were significantly lower compared with April (*Z* = 19.15, *p* < 0.001) and August (*Z* = 7.67, *p* < 0.001), indicating an increased proportion of nitrogen in the diet (figure 3, 5A,B). Finally, intake C : N was significantly higher during early summer (April) than that in August (*Z* = 11.48, *p* < 0.001, figure 3 and figure 5A).

Faecal C : N also varied significantly across seasons (*χ*^2^ = 36.64, *p* < 0.001, figure 5B). Faecal C : N in April was significantly lower compared with June (*Z* = 5.69, *p* < 0.001) and October (*Z* = 4.07, *p* = 0.001) and marginally lower compared with August (*Z* = 0.91, *p* = 0.803; figure 5B). Faecal C : N was significantly higher in June as compared with April and August (*Z* = 3.08, *p* = 0.017). While the faecal C : N in June was higher than that in October (*Z* = 1.63, *p* = 0.376), the difference was not statistically significant. Faecal C : N did not differ significantly between August and October (*Z* = 1.94, *p* = 0.224, figure 5B). See electronic supplementary material for all the pairwise comparisons for total carbon intakes, total nitrogen intakes, intake C : N and faecal C : N (electronic supplementary material, table S3).

## 4 Discussion

Animals are expected to adjust their foraging decisions to meet the nutritional requirements that maximize fitness within specific ecological contexts. Our results partially support the predictions for season-specific nutritional regulation in the Indian spiny-tailed lizard, *S. hardwickii*. We found that *S. hardwickii* adjusted the nutritional content of their diet across seasons in ways that seem to match key life-history activities. These lizards increased nitrogen intake in the season that coincides with their breeding period and carbon intake in the season just before winter hibernation. Our examination of faecal elemental composition also showed that the carbon to nitrogen ratios of lizard faeces varied across seasons, with greater C : N in June as compared with all other months. This indicates that these lizards can meet their nutritional requirements using both behavioural diet choice and post-ingestive nutrient retention.

Foraging decisions of animals can reflect resource availability in their habitat. Our results show that lizard diets constituted certain plant species more than expected by density-dependent foraging, suggesting that *S. hardwickii* have a strong preference for food plants across all seasons. Measurements of plant biomass across seasons showed that the plant species contributing the most to *S. hardwickii* diet was also the most abundant in the habitat (figure 2). However, the relative abundance of plant species was not commensurate with their relative abundance in lizard diet, suggesting that these lizards forage selectively and this pattern changed across seasons (figure 2). Dietary analysis of *S. hardwickii* intakes revealed a very narrow dietary breadth, wherein the lizards consumed the same three–four food items, comprising mostly a species of perennial grass, *Dactyloctenium* sp. and a forb, *Heliotropium* sp., across seasons, unlike agamid lizards of the closely related genus *Uromastyx* (from which *Saara* was recently separated [27]. Most studies on the feeding ecology of *Uromastyx* spp. report a wide range of plants in their diet, at least during the seasons when many plants are available [17,19,28]. However, in dry seasons, when fewer plant species are available, many herbivorous desert vertebrates, including *Uromastyx* spp. and *Saara* spp., rely on a very limited number of food plants to meet their nutritional needs. The choice of food plants in *S. hardwickii* might also reflect an avoidance of other plant species in the habitat that are better defended with secondary plant metabolites. However, further examination of plant metabolites and other nutrients is necessary to better understand the choice of food plants by these lizards.

Animal diet varies across seasons not only in its taxonomic composition, but also in nutritional content. By combining field observations of foraging behaviour with elemental analysis of dietary components, we found that nitrogen intake for *S. hardwickii* was highest in June. Although our analysis of resource use shows a preference for certain food plants, this focal increase in nitrogen intake cannot be explained by variation in plant consumption. Instead, we found that along with plants, *S. hardwickii* consumed significant amounts of insects in both June and August, constituting approximately 16–18% of the lizard diet by weight (figure 4). Compared with the main available food plants that consist of 1.68% N, insects contain a far higher concentration of nitrogen (11.20%). The timing of insect consumption by *S. hardwickii* coincides with their breeding season when gamete production may require elevated protein intake. Many animals, such as females of reindeer and caribou, consume high-protein food items during their pregnancy in the winter season [29]. In addition to field observations like these, reproductive activities have been linked to increased protein intake in tightly controlled laboratory experiments [30]. For *S. hardwickii*, termites were the main insects consumed, which we identified from the exoskeletal remains in the faecal pellets (M.J., unpublished). Although we do not know about seasonal variation in insect activity in the Thar desert, detritivores in other deserts are more active during dry seasons [31]. We speculate that the occasional pre-monsoon showers during June soften the fallen shrub branches, making them ideal for termite activity. *Saara hardwickii* was often found disturbing such termite-infested branches with their claws to consume these insects (M.J., personal observation). In April and October, insect consumption dropped to nearly 0%, even when insects were prevalent in the environment (M.J., personal observation). While some reports suggest a significant presence of animal matter in the diet of *U. aegyptia microlepis* [32], other studies on the same species report almost complete herbivory [28]. Notably, these two studies on *U. aegyptia microlepis* were carried out in different seasons. Similarly, a previous study on *S. hardwickii* reports negligible insect consumption from the same ecosystem as our study [19]. Given the strong seasonality associated with insect consumption that we report here, one possible explanation for the disparity in reports of diet composition of *Saara* spp. and *Uromastyx* spp. could be attributed to differences in the season in which field observations are made.

Seasonal variation in the nutritional content of the diet of *S. hardwickii* was also evident from a higher consumption of carbon in October. *Saara hardwickii* eats the same four species of plants all year round, but consumption of *Dactyloctenium* sp. is highest in this season (figure 2). Even though the per cent carbon content of this species was not greater than that of other available plants, *Dactyloctenium* sp. was the most abundant species in this season (electronic supplementary data; figure 2). Interestingly, as with increased nitrogen consumption in the breeding season, this peak in carbon consumption also coincided with a key life-history stage. *Saara hardwickii* hibernates in winter (from November to February) and is expected to build up stores of body fat before the start of winter. Animals across taxa are known to build up stores of body fat before the onset of hibernation [33,34], which are used during this inactive phase [35]. While diet composition associated with an increase in fat stores before hibernation is well studied in mammals [36], whether reptiles actively choose specific diet components to match the nutritional requirements of hibernation is not well understood.

Even though insects were available all year round, *S. hardwickii* shifted to a completely plant-based diet before hibernation in October, which enabled them to get closer to their intake target by obtaining high amounts of carbohydrate (carbon), without overconsuming nitrogen. This is important for animals since excess proteins can have detrimental effects on the body if not used [6]. In contrast, during the breeding season, the elemental composition of available plant species varied within a narrow range of C : Ns (electronic supplementary material, table S1). These lizards consume insects during this season, since accruing nitrogen (proteins) in sufficient amounts from these carbon-rich plants is not possible without an overconsumption of carbohydrates and plant fibre. Including a complementary food source rich in either carbon or nitrogen thus allows *S. hardwickii* to increase the consumption of required macronutrients without overconsuming others.

Variation in animal diet across seasons can reflect both active nutritional regulation and availability of nutritional resources. For example, giant pandas, *Ailuropoda melanoleuca*, eat bamboo shoots when they require nitrogen and phosphorus and eat leaves when they specifically need calcium and carbon, highlighting a dietary choice driven by physiological needs and not resource availability [37]. Grey-cheeked thrushes, *Catharus minimus*, that consume both insects and fruits, include more fruits during the migratory season compared with the breeding season, despite the availability of insects throughout the year [38]. The diet of Turpan wonder geckos, *Teratoscincus roborowskii*, includes more insects in spring when they reproduce, but more berries in autumn, suggesting a dietary choice influenced by both availability and physiological needs [39]. In *S. hardwickii*, nutritional intakes are similar across days in each season as compared with intakes across seasons, indicating that the lizards forage to occupy a relatively small area in the nutrient state space within seasons (figure 5A). We also found that the nutritional intakes were very similar in patches that varied in their resource abundance in a single season, indicating that these lizards choose specific ratios of C : N even when available plant resources are varying (electronic supplementary material, figure S3). This close aggregation of intakes in nutrient state space along with specific dietary choices across seasons suggests an active diet choice in these lizards to get closer to their seasonal nutritional needs. Although diet studies on reptilian species might hint at nutritional regulation (such as [39], but see [40]), they do not explicitly quantify any nutritional aspect of diet. Here, we provide a rare example of nutritionally explicit foraging in a lizard that shifts seasonally with varying nutritional needs and available resources.

*Saara hardwickii* can meet their nutrient requirements by regulating not just their intakes, but also by modulating digestion to retain specific nutrients differentially. We found that the carbon and nitrogen content of lizard faecal matter differed significantly between seasons. While lower faecal %C and %N in June compared with other seasons suggest that both the elements are retained more, a higher faecal C : N in this season reveals a relatively greater retention of nitrogen than carbon (figure 5B). Such selective retention of nutrients has been recently shown in the Peninsular rock agama, *Psammophilus dorsalis*, in which faecal C : N did not change but both carbon and nitrogen were retained more during the breeding season [40]. Such changes in faecal composition can be due to the action of several post-ingestive processes. Studies across taxa have shown that the concentration of digestive enzymes in gastric content is influenced by the composition of consumed macronutrients [41,42]. Changes in digestive enzymes enable animals to absorb and retain different macronutrients differentially, which is reflected in the faecal macronutrient composition [41]. While variation in faecal composition makes sense only in relation to the nutritional intakes of animals, very few studies have measured both dietary and faecal compositions in free-ranging animals (e.g. [7]). We found that the elemental composition of faecal matter and diet in June and August is relatively similar (figure 5B). In August, which is the rainy monsoon season, diet components are nutrient dense as compared with other seasons (indicated by carbon and nitrogen content of the consumed plants), potentially resulting in higher %C and %N of diet as well as faecal matter (electronic supplementary material, table S1; figure 5B). In October, elemental analysis of faecal matter revealed a higher nitrogen content than that of diet (i.e. lizards excrete a greater percentage of nitrogen than they consume), which suggests that they may be retaining more carbon. Thus, our results suggest that these lizards use post-ingestive regulation of nutrients in combination with dietary choices to meet their nutritional needs across seasons.

In conclusion, while many studies on free-ranging vertebrates support the idea that animals can sense and meet their transient as well as long-term nutritional demands, such studies are rarely carried out in animals inhabiting extreme habitats. Some studies have focused on animals in extremely cold seasonal habitats in temperate regions [9,10], but we still have little understanding of the nutritional ecology of desert vertebrates, especially reptiles. Our study fills this gap in the literature by examining the elemental composition of diet and faecal matter across four seasons in a desert agamid, the Indian spiny-tailed lizard. Our analysis of elemental ratios as a proxy for macronutrients suggested that *S. hardwickii* can meet its expected nutritional demands across seasons through selective ingestion of available food resources, as well as with the support of post-ingestive retention of nutrients. Our results also challenge the existing notion that these lizards are predominantly herbivorous, since we find a significant consumption of insects during the peak and post-breeding seasons. Overall, our study makes a case for examining nutrient-specific responses in vertebrates that inhabit extreme environments to understand the constraints animals face while foraging optimally to meet their season-specific nutritional requirements.

## Supplementary Material

Supplementary material is available online at https://doi.org/10.6084/m9.figshare.c.8213843.

Supplementary Material

## Data Availability

All data presented in this manuscript are available from the Dryad Digital Repository [43]. Supplementary material is available online [44].
