# Supplementary material for: Desert lizards modulate nutritional responses to match seasonal biological needs
Source: R Soc Open Sci. Author manuscript; Available in PMC 2026 Mar 4. (PMC7618820; doi:10.1098/rsos.251690)
Supplement: Supplementary Material [file EMS212582-supplement-Supplementary_Material.docx]

**Supplementary Tables**

**Supplementary Table 1:** Elemental composition (%C, %N, and C:N) of all the main dietary components across four seasons. The table contains all food resources that contributed ≥ 1% to the *S. hardwickii* intakes by mass in at least one season.

| **Season** | **Dactyloctenium** | | | **Euphorbia** | | | **Heliotropium** | | | **Aerva** | | | **Insects** | | |
| --- | --- | --- | --- | --- | --- | --- | --- | --- | --- | --- | --- | --- | --- | --- | --- |
|  | %C | %N | C:N | %C | %N | C:N | %C | %N | C:N | %C | %N | C:N | %C | %N | C:N |
| **April** | 11.27 | 0.85 | 13.26 | 27.55 | 2.29 | 12.04 | 18.28 | 1.27 | 14.37 | 40.77 | 3.17 | 12.87 | - | - | - |
| **June** | 11.47 | 0.88 | 13.03 | - | - | - | 17.78 | 1.2 | 14.77 | 37.74 | 2.96 | 12.76 | 52.8 | 11.2 | 4.71 |
| **August** | 42.08 | 3.32 | 12.68 | 39.16 | 2.37 | 16.56 | 16.65 | 0.95 | 17.59 | 39.9 | 3.78 | 10.57 | 49.9 | 10.9 | 4.58 |
| **October** | 35.81 | 2.08 | 17.19 | 26.99 | 2.02 | 13.33 | 34.73 | 2.39 | 14.54 | 39.64 | 2.3 | 17.26 | - | - | - |

**Supplementary Table 2**: Percentage contribution of each dietary component to the total carbon, total nitrogen, and total dietary mass of *S. hardwickii* intakes across seasons

| **Season** | **Dactyloctenium** | | | **Euphorbia** | | | **Heliotropium** | | | **Aerva** | | | **Insects** | | |
| --- | --- | --- | --- | --- | --- | --- | --- | --- | --- | --- | --- | --- | --- | --- | --- |
|  | Carbon | Nitrogen | Mass | Carbon | Nitrogen | Mass | Carbon | Nitrogen | Mass | Carbon | Nitrogen | Mass | Carbon | Nitrogen | Mass |
| **April** | 24.69 | 23.86 | 40.96 | 49.23 | 52.34 | 34.63 | 20.48 | 18.26 | 21.41 | 5.61 | 5.54 | 2.99 | - | - | - |
| **June** | 32.03 | 19.11 | 55.15 | - | - | - | 22.34 | 11.67 | 26.16 | 3.54 | 2.26 | 2.03 | 42.09 | 66.97 | 16.66 |
| **August** | 39.43 | 30.1 | 34.56 | 23.99 | 14.76 | 22.77 | 12.57 | 7.27 | 24.62 | - | - | - | 24.01 | 47.86 | 18.06 |
| **October** | 79.89 | 76.55 | 76.57 | 15.65 | 18.78 | 19.25 | 2.33 | 2.62 | 2.3 | 2.13 | 2.06 | 1.88 | - | - | - |

**Supplementary Table 3**: Results of the pairwise comparisons of total carbon intake amount, total nitrogen intake amount, intake C:N ratios, and faecal C:N ratios.

| **Comparison** | **Total Carbon** | | **Total Nitrogen** | | **Intake C:N** | | **Faecal C:N** | |
| --- | --- | --- | --- | --- | --- | --- | --- | --- |
|  | **t-ratio** | **p-value** | **t-ratio** | **p-value** | **t-ratio** | **p-value** | **t-ratio** | **p-value** |
| **April – August** | 3.416 | 0.0055 | 0.103 | 0.9996 | 11.484 | < 0.0001 | -0.905 | 0.8025 |
| **April – June** | -4.06 | 0.0007 | -13.255 | < 0.0001 | 19.151 | < 0.0001 | -5.693 | < 0.0001 |
| **April – October** | -11.298 | < 0.0001 | -4.418 | 0.0002 | -13.493 | < 0.0001 | -4.068 | 0.0011 |
| **August – June** | -7.476 | < 0.0001 | -13.358 | < 0.0001 | 7.666 | < 0.0001 | -3.079 | 0.017 |
| **August – October** | -14.714 | < 0.0001 | -4.522 | 0.0001 | -24.978 | < 0.0001 | -1.942 | 0.2238 |
| **June – October** | -7.239 | < 0.0001 | 8.837 | < 0.0001 | -32.644 | < 0.0001 | 1.625 | 0.3758 |

**Supplementary Figures**


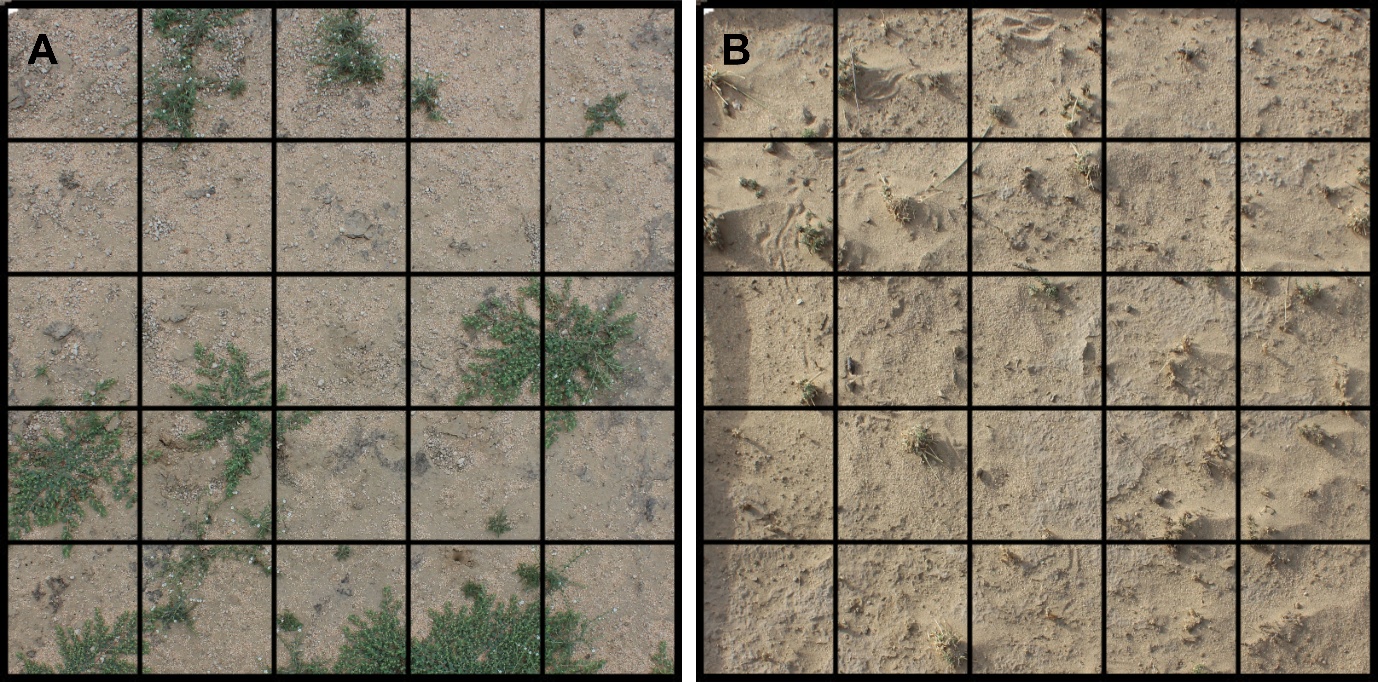


**Supplementary Figure 1**: Photographs of quadrats with superimposed Daubenmire frame for plant composition estimation during monsoon (A) and summer (B).


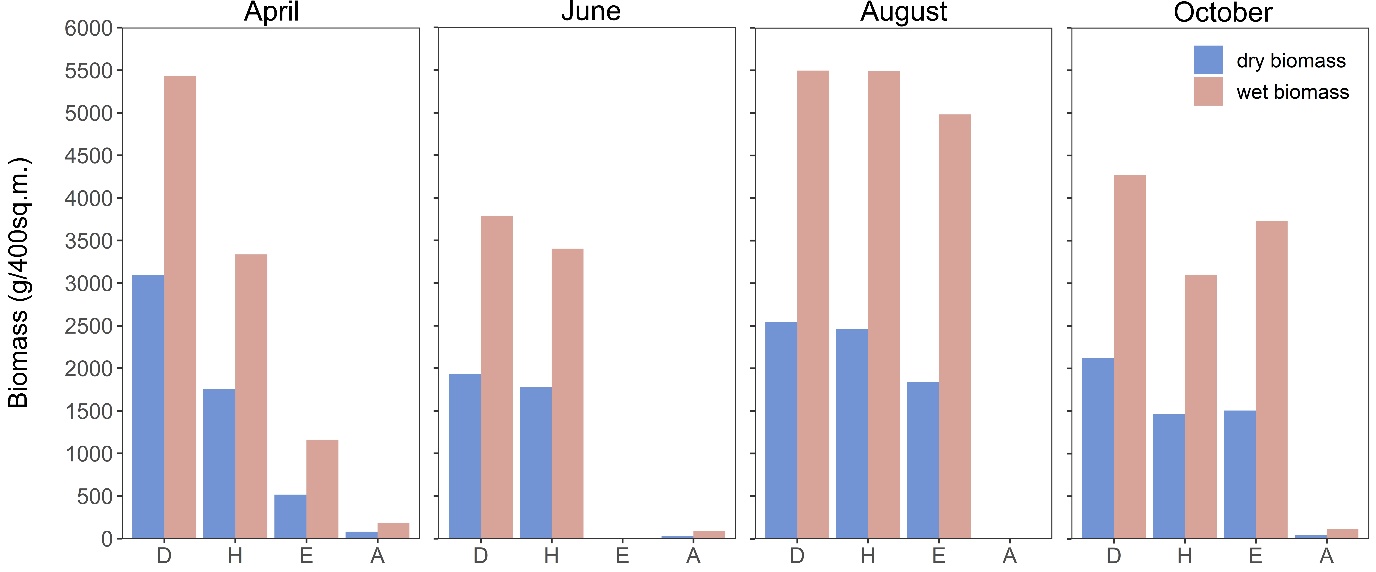


**Supplementary Figure 2**: Wet and dry biomasses of four main food plants of *S. hardwickii* across all the sampling seasons.


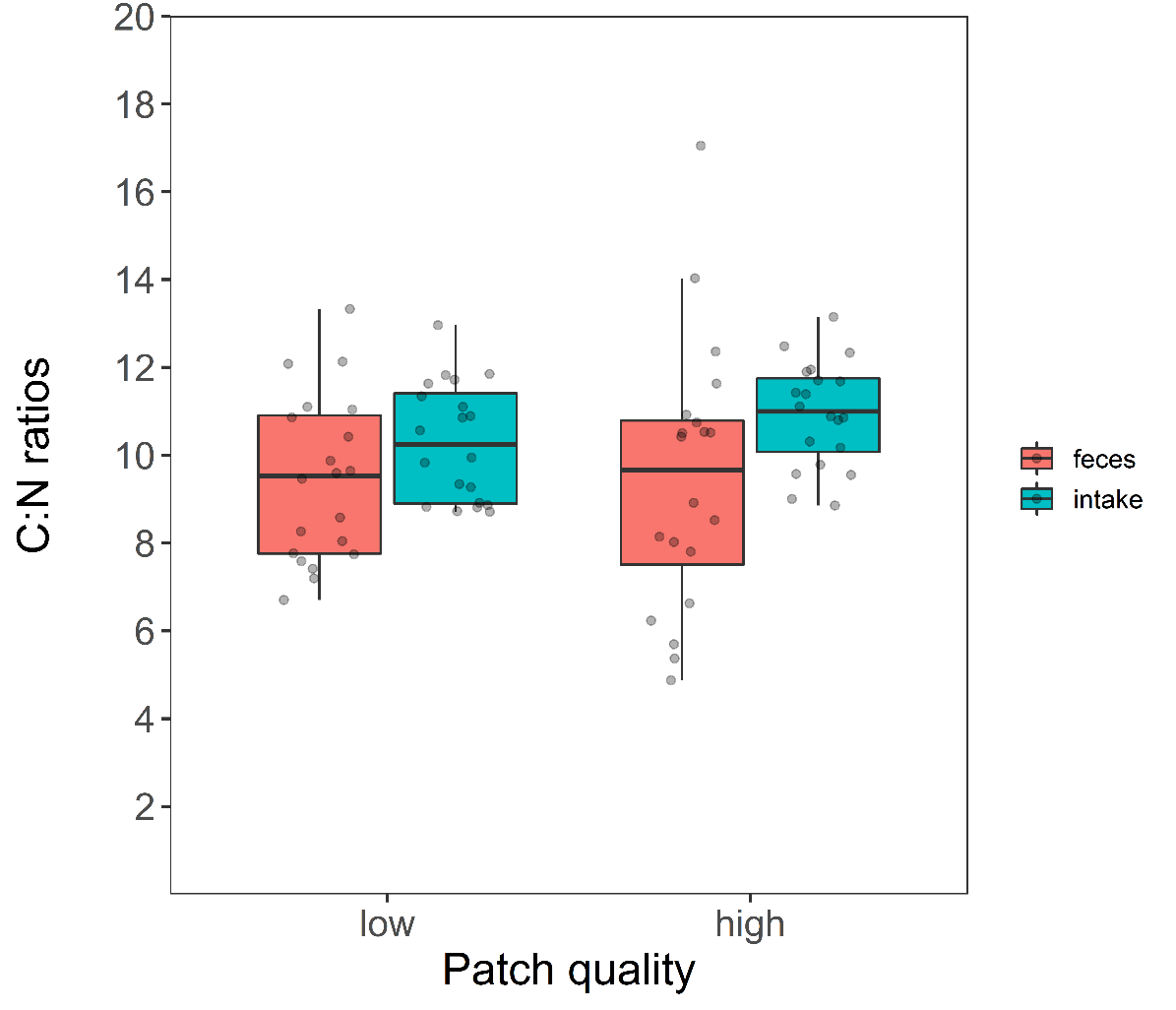


**Supplementary Figure 3**: Carbon:Nitrogen ratios of dietary intakes and fecal matter of *S. hardwickii* across patches varying in plant abundance. The boxplots represent the median C:N ratios with the interquartile range. Vertical lines represent quartile 1–1.5 × IQR and quartile 3 + 1.5 × IQR.
